# Carbonic Acid in Dental Practice

**Published:** 1901-06

**Authors:** William H. Potter


Carbonic Acid in Dental Practice.[1]—In this paper carbonic acid gas is considered as a means for the removal of sensibility from dentine. The two theories as to the cause of sensitiveness in dentine are first fully stated. One theory assumes the existence of nerve-fibres in the contents of the dentinal tubules, and the other theory holds that nerve-fibres reach no farther than to the region between the odontoblastic cells of the pulp, and that they are here acted upon in some way by the dentinal fibres. The author does not claim to settle the disputed point as to the reason why dentine is sensitive, but proceeds to a statement of the uses of carbon dioxide in general medicine. These he states to be to produce relief from pain in carcinoma of the breast and uterus, and other tumors, and in neuralgia of the uterus, the effect lasting from one-half to one hour. Carbon dioxide is also used as a disinfectant in bad-smelling discharges from wound surfaces, in catarrh of the vagina, in chronic skin diseases, ozæna, rheumatic affections, and other illnesses. In regard to the disinfection power of carbon dioxide exact investigations have been made by Kolbe.

In regard to the use of carbon dioxide in dentistry, the author says that he has given much time to the construction of an apparatus which makes it possible to apply the gas to dentine for the purpose of removing its sensibility.

His apparatus, which is made by Louis H. Loewenstein, Berlin, consists of three parts,—1. A carbon dioxide bottle filled with compressed gas. To it are added a reduction-valve and steam-gauge.

---

[1] Die Kohlensäure in der Zahnheilkunde, von Zahnarzt Max Bauchwitz, Stettin. Deutsche Monatsschrift für Zahnheilkunde, März, 1901.

2. A warming apparatus. 3. A small mixing-tube, by means of which carbon dioxide can be mixed with a medicament.

By this apparatus the gas goes from the storage bottle at a pressure of not over one-half atmosphere and enters the heating device, and is then discharged upon the tooth at the body temperature. The anæsthetic effect can be increased by the addition of a twenty per cent. cocaine solution. This solution is mixed with the carbon dioxide in the mixing-tube. The author's rule is to use first the pure gas, then the medicated gas, and finally pure gas, and anæsthesia is claimed in from two to three minutes.

It is interesting to note how the author explains the action of the carbonic acid gas in view of the two theories first stated in regard to the cause of the sensitiveness of dentine. Provided the dentinal fibrils contain no nerve-filaments, then the carbonic dioxide acts by impregnating the protoplasm of the dentine, and by its presence paralyzing it so that it conveys no impressions to the nerves of the pulp. If we allow that the dentinal fibrils contain nerve-fibres, then the carbon dioxide acts directly upon them.

A further use of a warm stream of carbonic gas is stated to be for the drying and disinfection of root-canals. The author mentions its use in the extraction of teeth and small surgical operations in the mouth as a proper field for investigation.

When we consider what results can be accomplished in the removal of sensitiveness from dentine by the use of warm air alone, it would seem that special pains should be taken with the apparatus just described, to be sure that the results attained were really dependent on the peculiar action of carbonic acid gas, and could not have been produced by the drying effect of a current of warm air. The author brings out very clearly, however, the physiological proposition that carbon dioxide exerts a paralyzing effect upon protoplasm, and thus offers a plausible explanation of its action on the contents of the dentinal tubules.—William H. Potter.

